# Novel Interaction Mechanism of a Domain Antibody-based Inhibitor of Human Vascular Endothelial Growth Factor with Greater Potency than Ranibizumab and Bevacizumab and Improved Capacity over Aflibercept* [Fn FN2]

**DOI:** 10.1074/jbc.M115.691162

**Published:** 2016-01-04

**Authors:** Adam Walker, Chun-Wa Chung, Margarete Neu, Manish Burman, Thil Batuwangala, Gavin Jones, Chi-Man Tang, Michael Steward, Michael Mullin, Nadia Tournier, Alan Lewis, Justyna Korczynska, Vicky Chung, Ian Catchpole

**Affiliations:** From ‡ BioPharm Innovation,; § Molecular Discovery Research,; ¶ BioPharm Discovery, and; ‖ BioPharm Process Research, GSK Medicine's Research Centre, Stevenage, Herts SG1 2NY, United Kingdom

**Keywords:** angiogenesis, antibody engineering, drug action, protein-protein interaction, retinal degeneration, single-domain antibody (sdAb, nanobody), vascular endothelial growth factor (VEGF)

## Abstract

A potent VEGF inhibitor with novel antibody architecture and antigen binding mode has been developed. The molecule, hereafter referred to as VEGF dual dAb (domain antibody), was evaluated *in vitro* for binding to VEGF and for potency in VEGF-driven models and compared with other anti-VEGF biologics that have been used in ocular anti-angiogenic therapeutic regimes. VEGF dual dAb is more potent than bevacizumab and ranibizumab for VEGF binding, inhibition of VEGF receptor binding assays (RBAs), and VEGF-driven *in vitro* models of angiogenesis and displays comparable inhibition to aflibercept (Eylea). VEGF dual dAb is dimeric, and each monomer contains two distinct anti-VEGF domain antibodies attached via linkers to a human IgG1 Fc domain. Mechanistically, the enhanced *in vitro* potency of VEGF dual dAb, in comparison to other anti-VEGF biologics, can be explained by increased binding stoichiometry. A consistent model of the target engagement has been built based on the x-ray complexes of each of the two isolated domain antibodies with the VEGF antigen.

## Introduction

Anti-VEGF therapy is the current standard of care in the treatment of wet age-related macular degeneration (1). The required monthly (ranibizumab/bevacizumab) or bi-monthly, (aflibercept) intravitreal injections present both a risk and burden to patients (1, 2). There is, therefore, a need to explore less frequent dosing to reduce the safety risk and treatment burden (2, 3).

One approach is to develop a slow release formulation of an anti-VEGF molecule with a target product profile that is more amenable to patients and clinicians for administration approximately twice a year to address this burden (3).^3^ Such a system could also offer benefit against highly vascularized tumors such as glioblastoma (4). To develop such a system, a molecule with an improved binding capacity toward VEGF at low concentrations would be preferable.

In humans and other mammals, the VEGF family consists of five related glycoproteins: VEGF-A, -B, -C, and -D and placental growth factor (5). VEGF-A is the most well studied, and although encoded by a single gene, several distinct isoforms of VEGF-A exist as a result of alternative splicing and/or proteolytic cleavage. The various VEGF-A isoforms are all active as homodimers (6), differing principally in their size and their ability to bind heparin or accessory proteins called neuropilins (7, 8). VEGF-A_165_ is the predominant isoform expressed in humans, and VEGF-A_121_ is also expressed at high levels in many tissues and in pathological conditions (9) with isoforms of VEGF-B also produced by alternative splicing (10).

VEGF family ligands bind with high affinity to, and signal through 3 receptor tyrosine kinases, VEGF receptor 1 (VEGFR1),^4^ VEGFR2, and VEGFR3 (11–13). VEGFR1 is expressed on the vascular endothelium and also by several other cell types. VEGFR2 is expressed predominantly on vascular endothelial cells. VEGFR1 exhibits only weak tyrosine phosphorylation when activated by VEGF-A; thus the effects of VEGF-A isoforms on the vascular endothelium are thought to be mediated primarily through activation of VEGFR2 (11, 12).

Domain antibodies are the smallest (11–15 kDa) antigen binding fragments of immunoglobulins (14, 15). Domain antibodies tend to maintain their activity when present in protein fusions, which makes their use attractive in protein engineering approaches to develop novel biopharmaceutical modalities (14–16). We have developed the dual dAb architecture using VEGF dAbs fused to human IgG1 domain and demonstrated high affinity binding of VEGF dual dAb to human, mouse, rabbit, and pig VEGF. Cyno and rat VEGF sequences are identical to human and mouse VEGF-165/164, respectively, enabling evaluation of efficacy using preclinical models from lower species. The meso scale discovery (MSD) platform was used to investigate the binding of VEGF dual dAb to human VEGF family ligands VEGF-A_121_, VEGF-A_165_, VEGF-B_167_, VEGF-C, and VEGF-D and mouse VEGF-164. The *in vitro* potency of VEGF dual dAb was also measured in receptor binding inhibition assays (RBAs). In RBAs VEGF dual dAb was evaluated alongside ranibizumab and bevacizumab and found to be more potent, with comparable potency to aflibercept (VEGF Trap or Eylea). We have also demonstrated that Fc binding is maintained in the VEGF dual dAb format using the Proteon surface plasmon resonance (SPR) platform to investigate potential Fc interactions of VEGF dual dAb alone and when bound to human VEGF-165 by studying binding to C1q, FcRn, and Fcγ receptors (17–19).

Methods have been developed to measure the ability of endothelial cells to associate into tubules when in contact with various matrix proteins or cell types as an *in vitro* model for angiogenesis (20). To assess whether the more potent *in vitro* inhibition of VEGF receptor-ligand interactions by VEGF dual dAb might potentially translate to more potent inhibition of angiogenesis, we compared the efficacy of VEGF dual dAb with those of aflibercept, bevacizumab, and ranibizumab in the tubule formation assay (21–23). Comparable efficacy of VEGF dual dAb and aflibercept was demonstrated, whereas VEGF dual dAb shows superior efficacy to ranibizumab and bevacizumab in this assay system.

Stoichiometry of VEGF dual dAb binding to VEGF has been investigated using SEC-MALLS (size-exclusion chromatography multi-angle laser light scattering) analysis. The identification of the VEGF binding site on the VEGF dAbs by protein crystallography combined with molecular modeling suggests a novel mechanism of action. The VEGF dual dAb is unique in its ability to sequester two VEGF dimers per molecule, most likely by a “side-on” engagement mechanism, whereas other common VEGF inhibitors only engage with VEGF in multiples of a 1:1 interaction. The combined data set explains the improved capacity and potency of the VEGF dual dAb over current standard of care anti-VEGF molecules.

## Experimental Procedures

### 

#### 

##### Expression and Purification of VEGF Dual dAb Protein

VEGF dual dAb was PCR amplified and cloned into pDOM50, a derivative of the pTT5 HEK293E expression vector (National Research Council, Canada) using EcoRI/HindIII restriction sites. Protein was expressed in HEK293 cells and secreted into the culture supernatant (24). Expressed protein was then purified directly from clarified culture supernatant using Protein A Streamline resin (GE Healthcare) according to the manufacturer's instructions.

##### Expression and Purification of dAb Proteins

V_H_ and V_κ_ VEGF dAbs (25) were PCR-amplified and cloned into the PET30 expression vector (Novagen) and expressed in BL21 *Escherichia coli* after growth in TB OnEx auto-induction medium (Novagen) supplemented with 35 μg/ml kanamycin at 30 °C for 72 h. Expressed protein was purified from clarified culture supernatant using protein A and protein L Streamline resin, respectively.

##### Expression and Purification of Human VEGF_1–165_

Human VEGF_1–165_ was PCR-amplified and cloned into pDOM50. Protein was expressed in HEK293 cells as described above. Culture supernatant was then clarified by centrifugation and passed through a 0.2-μm filtration unit (Nalgene) before loading onto a heparin-Sepharose column (GE Healthcare) using an Akta HPLC system. Pooled VEGF-containing fractions were filtered and concentrated using spin dialysis units with a 10,000-Da molecular mass cut off, then purified using a HiLoad 26/60 Superdex 200 prep grade size exclusion column (GE Healthcare) equilibrated in PBS supplemented with 0.3 m NaCl and 10% sorbitol.

##### Expression and Purification of Human VEGF_1–107_·His_6_

Human VEGF_1–107_ with C-terminal His_6_ tag was PCR-amplified and cloned into pDOM50. Protein was expressed in HEK293 cells as described above. Culture supernatant was then clarified by centrifugation, and buffer exchanged into PBS using a prep scale Tangential Flow Filtration unit (Millipore). After buffer exchange into PBS the protein was passed through a 0.2-μm filtration unit (Nalgene) and loaded onto a 5-ml chelating Hitrap column (GE Healthcare) charged with NiCl_2_ as described in the manufacturer's guide, using an Akta HPLC system.

After loading, the column was washed with running buffer (100 mm Tris, 100 mm NaCl, pH 8) and eluted with a gradient of running buffer supplemented with 1 m imidazole. Protein-containing fractions were then pooled and dialyzed against running buffer to remove imadazole.

##### Comparator VEGF Inhibitor Molecules

Ranibizumab, (Lucentis) and bevacizumab (Avastin) were obtained from Roche Applied Science, and aflibercept (Eylea) was obtained from Bayer AG, (Leverkusen, Germany).

##### Human and Murine VEGF Binding to VEGF Dual dAb Captured on Protein A

Protein A was immobilized on a Biacore C1 chip (GE Healthcare) by primary amine coupling. VEGF dual dAb was captured on the protein A surface to a level of 20 resonance units. VEGF samples were diluted to 32, 8, 2, 0.5, 0.125, and 0.03 nm in HBS-EP buffer (GE Healthcare) and injected over the captured VEGF dual dAb at a flow rate of 60 μl/min. After each injection, the surface was regenerated using 50 mm NaOH, which allowed removal of protein A-bound VEGF dual dAb without affecting the protein A surface. The run was carried out at 37 °C with HBS-EP as the running buffer on a T200 Biacore experiment. The curves were double-referenced using a buffer-only curve, and the data were fitted to the 1:1 binding model inherent to the T200 evaluation software version 1.0. The 32 nm curves were removed from the data set before analysis to allow a better fit. Experiments were run in triplicate, and average values for association/dissociation rates (with S.D. calculated) and dissociation constants were determined using the triplicate set of data (Table 1).

**TABLE 1:** **VEGF dual dAb affinity for VEGF ligands from different species** Data are the mean from *n* = 3 experiments, determined by surface plasmon resonance (numbers in parentheses represent S.D.)

Ligand	*k_a_*/10^5^ m^−1^·s^−1^	*K_d_*/10^−5^ s^−1^	*K_D_*
			*pm*
Human VEGF-A_165_*^a,b^*	66.3 (6.38)	4.76 (0.70)	7.17
Human VEGF-A_165_*^a,c^*	81.3 (0.03)	8.08 (1.68)	9.94
Murine VEGF-A_164_*^a^*	80.0 (0.03)	5.19 (1.68)	6.50
Human VEGF-A_165_*^b,d^*	578.0 (105.9)	18.8 (2.51)	3.27
Human VEGF-A_165_*^c,d^*	347.0 (2.01)	10.9 (0.76)	3.14
Porcine VEGF-A*^b^*	122.0 (9.1)	4.83 (0.40)	4.01

*^a^* VEGF dual dAb captured by protein A.

*^b^* Mammalian cell line-derived.

*^c^ E. coli* expressed.

*^d^* Immobilized VEGF.

##### VEGF Dual dAb Binding to Immobilized Human and Porcine VEGF

Human (Peprotech) and porcine (MyBiosource) VEGF-165 and VEGF-164 proteins, respectively, were immobilized on a Biacore C1 chip by primary amine coupling. The level of VEGF immobilized on the chip surface was 100 resonance units in the case of human and 700 resonance units in the case of porcine. VEGF dual dAb was diluted to 64, 16, 4, 1, 0.25, and 0.0625 nm in HBS-EP buffer and injected over the immobilized antigens at a flow rate of 60 μl per min. After each injection, the surface was regenerated using 10 nm glycine at pH 2, which allowed removal of bound VEGF dual dAb without affecting the immobilized antigen. Experiments were carried out at 37 °C with HBS-EP as running buffer on a T200 Biacore instrument. The curves were double-referenced using a buffer only curve, and the data were fitted to the 1:1 binding model inherent to the T200 evaluation software version 1.0. The 64 nm curves were removed from the data set before analysis as the shape of the curve suggested some nonspecific binding at this concentration. Experiments were run in triplicate, and average values for association/dissociation rates (with S.D. calculated), and the dissociation constant was determined using the triplicate set of data (Table 1).

##### VEGF Dual dAb Binding to C1q

VEGF dual dAb alone or in complex with human VEGF-165 (as described below) were immobilized via primary amine coupling on a GLM Proteon Chip (Bio-Rad). C1q protein diluted in HBS-N (GE Healthcare) + 10 mm CaCl_2_ at 256, 64, 16, 4, and 1 nm was injected over the immobilized antibody. A 0 nm (buffer only) was used for double-referencing the curves. Experiments were carried out at 25 °C in HBS-N + 10 mm CaCl_2_ as the running buffer. The data were analyzed using the Proteon Manager software version 3.1.0.6. *K_D_* values were generated for comparison of dual dAb alone or in complex with VEGF when possible using the equilibrium model (Table 2).

**TABLE 2:** **Demonstration of VEGF dual dAb binding to human Fc receptors** Data were generated by surface plasmon resonance (numbers in parentheses represent S.D.)

Ligand	*K_D_*
	*nm*
Human C1q	7.51
Human C1q*^a^*	14.0
Human FcRn	325.0
Human FcRn*^a^*	479.0
Human FcγRI / CD64	6.55
Human FcγRIIa (H isoform)	95.0
Human FcγRIIa (R isoform)	95.4 (13.6)*^b^*
Human FcγRIIb/c (CD32b/c)	274.0
Human FcγRIIIa (CD16a)	40.9
Human FcγRIIIa (V212)	36.9
Human FcγRIIIaR (17–208-F21)	114.0
Human FcγRIIIb (CD16b)	41.5

*^a^* Isolated complex with VEGF pre-bound.

*^b^* Mean value from two separate FcγRIIa protein batches.

##### VEGF Dual dAb Binding to FcRn

VEGF dual dAb alone or in complex with human VEGF-165 (as described below) were immobilized via primary amine coupling on a GLM Proteon Chip. The run was carried out at 25 °C either in HBS-EP, pH 7.4 or pH 6, as the running buffer. FcRn protein diluted in HBS-EP, pH 7.4 or pH 6, at 1000, 250, 62.5, 15.6, and 3.9 nm was injected over the immobilized antibody. A 0 nm (buffer only) was used for double-referencing the curves. The data were analyzed using the Proteon Manager software version 3.1.0.6. *K_D_* values were generated for comparison when possible using the equilibrium model (Table 2).

##### VEGF Dual dAb Binding to FcRγ

Anti-His tag antibody was immobilized to a GLM proteon Chip via primary amine coupling. His-tagged Fcγ receptors were captured on this surface. VEGF dual dAb alone or in complex with human VEGF-165 (as described below) were diluted at 500, 125, 31.2, 7.8, and 1.95 nm in HBS-EP buffer and injected over the captured receptor. A 0 nm (buffer only) was used for double-referencing the curves. The data were analyzed using the Proteon Manager software version 3.1.0.6. *K_D_* values were generated when possible using the equilibrium model (see Table 2) to verify binding.

##### VEGF Dual dAb Antigen Binding by MSD

A MSD high bind 96-well plate was coated with 2 μg/ml human VEGF-A_165_ (purified as described above), VEGF-A_121_, VEGF-B_167_, VEGF-C, VEGF-D, or mouse VEGF-164 (all from R&D Systems) in PBS, and the sealed plates were incubated overnight at 4 °C. The following day the plates were washed with Tris wash buffer (MSD) and blocked with 3% BSA in PBS for 1 h at room temperature. Plates were washed as described above before the addition of standard concentrations of VEGF dual dAb diluted in 0.1% BSA in PBS and incubated at room temperature for 2 h. Plates were washed again before the addition of the detection reagent (in-house sulfo-TAG-labeled goat anti-human IgG, Fc-specific; Sigma) at 1 μg/ml in 1% BSA in PBS and incubated at room temperature for 1 h. Plates were washed, and 2× Read Buffer T (MSD) was added before being read on a SECTOR 6000 MSD imager. GraphPad Prism was used to fit curves using a sigmoidal dose response (variable slope) model and to calculate the EC_50_ values, (with S.D. calculated over the mean) from *n* = 3 experiments.

##### Inhibition of VEGF Binding to VEGF Receptor

A MSD standard bind 96-well plate was coated with 0.25 μg/ml VEGFR1 or VEGFR2 (R&D Systems) in bicarbonate buffer, and sealed plates were incubated overnight at 4 °C. The following day plates were washed with Tris wash buffer and blocked with 3% BSA in PBS for 1 h at room temperature. Plates were washed as described above before the addition of anti-VEGF molecule and incubated at room temperature for 10 min before the addition of VEGF-165 (R&D Systems). The anti-VEGF molecules and VEGF were diluted in 0.1% BSA in PBS. The final VEGF concentration used was 10 ng/ml. No VEGF and VEGF-only wells were also included as controls on all plates. Plates were incubated at room temperature for 2 h before being washed again before the addition of the detection reagent (goat anti-human VEGF biotinylated antibody; R&D Systems) at 0.25 μg/ml in 1% BSA in PBS and incubated at room temperature for 1 h. Plates were washed again before the addition of the streptavidin sulfo-TAG (MSD) at 2 μg/ml in 1% BSA in PBS and incubated at room temperature for 30 min. Plates were washed, and 2× Read Buffer T was added before being read on a SECTOR 6000 MSD imager. GraphPad Prism was used to fit curves using a sigmoidal dose response (variable slope) model and to calculate the IC_50_ values (with S.D. calculated over the mean) from *n* = 2–11 experiments.

##### Inhibition of VEGF-dependent Tubule Formation

Tubule formation was assayed using the V2a angiogenesis assay (TCS Cellworks) as described in the manufacturer's protocol. V2a Co-culture cell aliquots were thawed into complete seeding medium (TCS Cellworks), transferred to 24-well plate culture wells, and incubated at 37 °C with 5% CO_2_ in a humidified environment. The following day test compounds were diluted with Dulbecco's PBS to a working stock concentration of 5.23 μm and stored at 4 °C. Positive (VEGF) and negative (suramin) control compounds (TCS Cellworks) were diluted into complete optimized growth medium (TCS Cellworks) to 2 ng/ml and stored at 4 °C. Working stock concentrations of test compounds were diluted into optimized growth medium with or without 2 ng/ml VEGF. Medium was aspirated from cultured cells, replaced with 0.5 ml of optimized growth medium ± control or test compound, and cells were returned to incubation. Medium was removed from cultured cells and replaced with fresh media ± control or test compounds (freshly prepared from working stock) on days 4, 6, 9, and 11. Medium was aspirated from plate wells on day 14, and cells were washed with Dulbecco's PBS then fixed by the addition of ice-cold 70% ethanol and incubated for 30 min at ambient temperature. Cells were washed 3 times with 1% BSA in Dulbecco's PBS (blocking solution), then incubated in a 1/400 dilution of anti-human CD31 mouse primary antibody (TCS Cellworks) at 37 °C for 60 min. Cells were washed as before then incubated in a 1/500 dilution of alkaline phosphatase-conjugated goat anti-mouse antibody (TCS Cellworks) at 37 °C for 60 min. Cells were washed three times with water.

For anti-CD31 ELISA analysis cells were incubated in 0.3 ml of 1 mg/ml *para*-nitrophenyl phosphate in 0.2 m Trizma base (Tris base), pH 9.8 (Sigma) at 37 °C for 20 min. Two 0.1-ml aliquots were removed from each sample, and each was added to 25 μl of 3 m NaOH in a 96-well ELISA plate. 0.1 ml of *para*-nitrophenyl phosphate solution was used in duplicate as a negative control. The absorbance at 405 nm of sample wells was measured using a SpectraMax M5e plate reader.

For image analysis the remaining *para*-nitrophenyl phosphate solution was removed, and cells were washed 3 times with water. Cells were incubated in 5-bromo-4-chloro-3-indolylphosphate)/*para*-nitrotetrazolium blue, solution (Sigma) for 10–15 min until staining developed. Supernatant was decanted from sample wells, and plates were washed three times with water and air-dried. Three representative images from each sample well were captured using a 4× objective lens. Images were analyzed using Angiosys software: tubules were automatically highlighted then manually edited to trace the path of each tubule, highlights were skeletonized to reduce the image to lines following the tubules, and then tubule data (total length, average length, number of tubules, and number of junctions) was captured. Variations in staining intensity occasionally caused inconsistencies in tubule measurements (length, number of tubule junctions) so the total tube length in each image was used as the most accurate and robust measure of tubulogenesis for further analysis.

##### VEGF Dual dAb-VEGF_1–165_ and Bevacizumab-VEGF_1–165_ Complex Preparation

VEGF dual dAb was used to generate a complex with human VEGF_1–165_ by mixing the two in various molar ratios (VEGF dual dAb-VEGF, 1:1, 1:2, 2:1, and 4:1) as follows. VEGF dual dAb was diluted 1:10 in 20 mm sodium acetate, 4% sucrose, 0.5% glycine, pH 5.5. VEGF was added, and the antigen-antibody complex was allowed to form by incubating at room temperature for 1 h. After clarification using a 0.2-μm filter the VEGF dual dAb-VEGF complex was purified on a HiLoad 26/60 Superdex 200 prep grade size exclusion column. 2-ml fractions were collected and analyzed by non-reducing SDS-PAGE using 4–12% Bis-Tris gradient gels run in 1× MES SDS-PAGE buffer. Fractions eluting in the first visible peak were pooled and concentrated using a Vivaspin 20 spin dialysis unit (Sartorius-Stedim). Concentrated protein was analyzed by SDS-PAGE, and protein concentration was determined by UV280 spectrophotometry. Samples were aliquoted before snap-freezing on dry ice. Bevacizumab complexes with VEGF_1–165_ were generated similarly.

##### SEC-MALLS Analysis

Shimadzu LC-20AD Prominence was used in series with mini DAWN TREOS and Optilab REX for this study. The samples were run through 20 mm sodium acetate, 4% sucrose, 0.5% glycine, pH 5.5, as the mobile phase at a flow rate of 0.5 ml/min. 100 μl of the dual dAb-VEGF complex was injected on a TSKGel G3000 SW_XL_ column (7.8 mm × 300 mm), where molecules were subjected to separation by hydrodynamic volume. The data were then analyzed using Astra 6.1.1.17 (see Fig. 4*A*). For analysis of input, sub- and super-stoichiometric complexes of the dual dAb-VEGF, (1:2, 2:1, and 4:1) and bevacizumab-VEGF, (1:1, 1:2, 2:1, and 4:1). The samples were run through PBS, pH 7.4, as the mobile phase at a flow rate of 0.5 ml/min. 100 μl of complex was injected on a Superdex TM200 SW_XL_ column (10 mm × 300 mm), where molecules were subjected to separation by hydrodynamic volume. The data were then analyzed using Astra 6.1.1.17, (see Fig. 4, *A* and *B*).

##### VEGF_1–107_·His_6_/dAb Complex Preparation

VEGF_1–107_·His_6_and VEGF-specific dAb (V_κ_ or V_H_) were used to generate a complex by mixing the two in a 1:2.5 molar ratio (VEGF_1–107_·His_6_/dAb complex) and incubated for 1 h at room temperature. After clarification using a 0.2-μm filter, complexes were purified on a HiLoad 26/60 Superdex 200 prep grade size exclusion column. After elution from the column 2-ml fractions were collected and analyzed by SDS-PAGE using 4–12% Bis-Tris gradient gels run in 1× MES SDS. Fractions shown to contain both dAb and VEGF_1–107_·His_6_ were then pooled and concentrated using Vivaspin 20 spin dialysis units. The concentrated samples were analyzed by SDS-PAGE as described above. Samples were then diluted to 10 mg/ml and snap-frozen on dry ice.

##### Crystallization of VEGF/V_κ_·dAb Complex

Crystals of the hVEGF_1–107_·His_6_/V_κ_·dAb complex were obtained using the vapor diffusion method within a MRC plate. Sitting drops contained a 1:1 ratio of well solution consisting of 20% PEG3350, 0.1 m Bis-Tris propane, pH 8.5, 0.2 m sodium citrate, and protein complex at 10 mg/ml at 4 °C. Crystals were harvested into a cryo-loop and briefly transferred into a cryoprotectant buffer of 20% glycerol and 80% well solution before being plunge-frozen in liquid nitrogen. Data from single crystals were collected at 100 K on the I04-1 beamline at the Diamond Light Source (Harwell). Diffraction was typically weak and anisotropic, with the degree of anisotropy varying between crystals. Data processing was achieved using XDS (26) within AUTOPROC (Global Phasing Limited) and scaled using SCALA (27) within the CCP4 programming suite (28). Structure solution was successful with a dataset from a single crystal with low anisotropy and good completeness to 2.7 Å. The I222 space group with cell dimensions of *a* = 61.221 Å, *b* = 147.128 Å, *c* = 175.171 Å, and α = β = γ = 90.00° was solved by molecular replacement using PHASER (29) via CCP4 and truncated models of 1VPP.pdb (VEGF) and 3EO9.pdb. The successful solution had one VEGF dimer and two domain antibodies within the asymmetric unit, resulting in a high solvent content of 67.6%. Iterative rounds of model building were performed using COOT (30). Refinement used REFMAC within the CCP4 programming suite (31). The statistics for data collection and final coordinates are given in supplemental Table 1 and sample electron density maps in supplemental Fig. 1. The crystal structure is deposited in the Protein Data Bank with entry code 5FV1.

##### Crystallization of VEGF/V_H_·dAb Complex

Crystals of the hVEGF_1–107_·His_6_/V_H_·dAb complex were obtained using the vapor diffusion method within a MRC plate at 20 °C. Sitting drops contained a 1:1 ratio of well solution consisting of 0.1 m Tris, pH 8.5, 7% PEG6000, 0.2 m MgCl_2_, and protein complex at 13.7 mg/ml. Crystals were harvested into a cryo-loop and briefly transferred into a cryoprotectant buffer of 30% ethylene glycerol and 70% well solution before being plunge-frozen in liquid nitrogen. Data from single crystals were collected at 100K on the I04-1 beamline at the European Synchrotron Radiation Facilities (Grenoble). Diffraction was again weak and anisotropic, with the degree of anisotropy varying between crystals. Data processing was achieved using MOSFLM (32) and SCALA within the CCP4 programming suite. Structure solution was successful by molecular replacement with a 3.45 Å dataset with a C2 space group with cell dimensions of *a* = 107.108 Å, *b* = 130.397 Å, *c* = 81.178 Å, α = γ = 90.00, and β = 106.53°. Molecular replacement using PHASER via CCP4 and truncated models of 1VPP.pdb (VEGF) and 1T2J.pdb (V_H_·dAb). The final structure had three VEGF and three domain antibody chains within the asymmetric unit. These formed one complete complex consisting of a VEGF dimer with a domain antibody at either end of the dimer, with the single remaining VEGF and antibody chain forming a crystallographic dimer across the C2 symmetric axis. Iterative rounds of model building and refinement were performed using COOT and REFMAC within the CCP4 programming suite.

The statistics for data collection and final coordinates are given in supplemental Table 1 and sample electron density maps in supplemental Fig. 1. The crystal structure is deposited in the Protein Data Bank with entry code 5FV2.

##### Modeling of VEGF Engagement with VEGF Dual dAb Molecule

Structural models of the dual dAb molecule in complex with VEGF in various orientations were generated from the two VEGF-dAb co-crystal structures and the Fc domain from the human IgG1 crystal structure (Protein Data Bank (PDB) code 1HZH; Ref. 33) using MOE 2013.0802 (Molecular Operating Environment, Chemical Computing Group Inc., Montreal, QC, Canada).

##### Comparative Binding Modes of Common VEGF Inhibitors to VEGF

**FIGURE 1.**
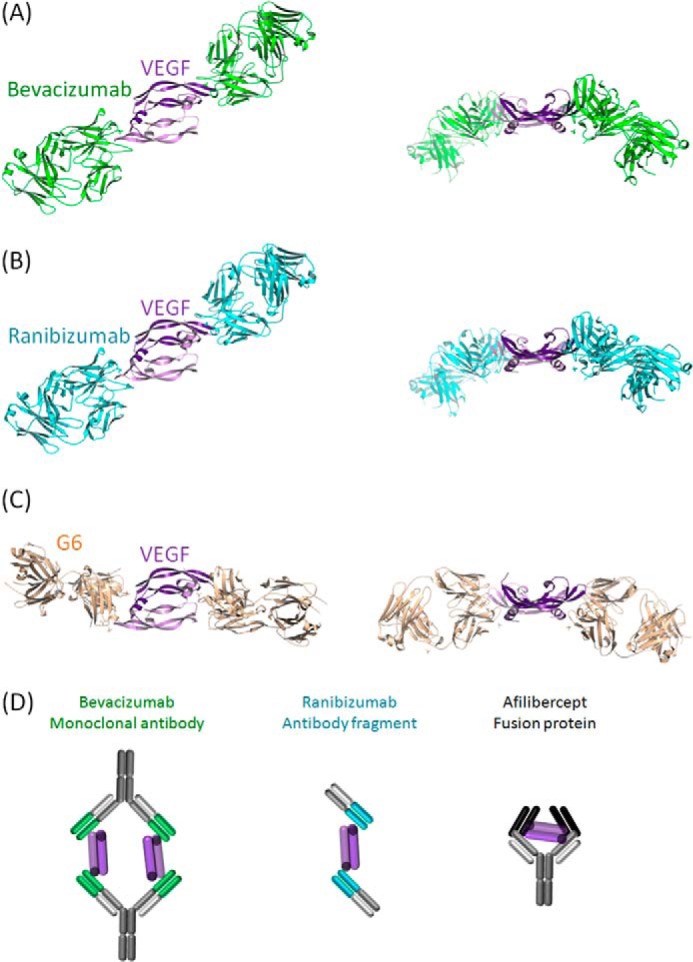
**Experimental x-ray coordinates from the PDB for VEGF engagement of bevacizumab (Avastin, PDB code 1BJ1) (*A*), ranibizumab (Lucentis, PDB code 1CZ8) (*B*), and G6 antibody (PDB code 2FJG) (*C*) and schematic models of VEGF engagement for bevacizumab, ranibizumab, and aflibercept (Eylea/VEGF trap) (*D*) (34, 37, 38).**

Both experimental x-ray coordinates from the Protein Data Bank for the VEGF engagement of ranibizumab (Lucentis, PDB code 1CZ8) (*A*), bevacizumab (Avastin PDB code 1BJ1) (*B*), G6 antibody (PDB code 2FJG) (34) (*C*), and schematic models of VEGF engagement with bevacizumab, ranibizumab, and aflibercept (Eylea/VEGF trap); structure not published in PDB (*D*), are shown in Fig. 1.

## Results

### 

#### 

##### High Affinity VEGF Binding

The cross-reactivity of VEGF dual dAb with human mouse and porcine VEGF species was investigated on the Biacore platform, and VEGF dual dAb was found to bind all proteins with similar affinity (see Table 1) where the data generated is a mean from a set of three repeats within the same experiment.

The binding of VEGF dual dAb to VEGF family and isoforms was investigated using the MSD platform. VEGF dual dAb bound to human VEGF-A_165_, human VEGF-A_121_, and mouse VEGF-164 with a higher affinity (lower EC_50_) than ranibizumab (Lucentis), bevacizumab (Avastin), and aflibercept (Eylea/VEGF Trap); see Table 3 where the data generated is the mean from a set of three repeats. VEGF dual dAb was found not to bind to human VEGF-B_167_, human VEGF-C, and VEGF-D compared with control proteins, which were known to bind these VEGFs (data not shown).

**TABLE 3:** **Comparative binding affinities of VEGF dual dAb and aflibercept to different human VEGF isoforms** Data are the mean from *n* = 3 experiments, determined by MSD (numbers in parentheses represent S.D.).

Molecule	Ligand	EC_50_
		*pm*
Aflibercept	Human VEGF-A_165_	86 (10.3)
VEGF dual dAb	Human VEGF-A_165_	32 (2.7)
Aflibercept	Human VEGF-A_121_	317 (83.0)
VEGF dual dAb	Human VEGF-A_121_	127 (22.1)
Aflibercept	Murine VEGF-A_164_	95 (20.2)
VEGF dual dAb	Murine VEGF-A_164_	36 (4.7)

##### Inhibition of VEGF Ligand Binding to VEGF Receptor

**FIGURE 2.**
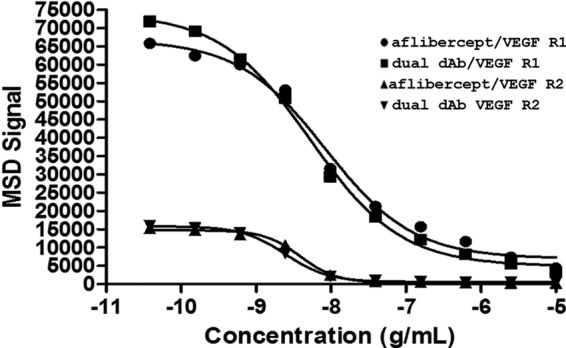
**Activity of VEGF dual dAb and aflibercept in receptor binding assays.** Inhibition of VEGF binding to VEGFR1 by aflibercept (●) or VEGF dual dAb (■) and VEGF binding to VEGFR2 by aflibercept (▴) or VEGF dual dAb (▾) was measured by MSD. Representative data are from one experiment (*n* = 3 similar experiments performed; see Table 4).

Receptor binding assays were used to investigate the ability of anti-VEGFs to prevent human VEGF-A_165_ binding to its receptors. VEGF dual dAb IC_50_ values were lower than those obtained with ranibizumab (Lucentis) and bevacizumab (Avastin) but only slightly lower than those obtained with aflibercept (Eylea) in receptor binding assays using VEGFR1 and VEGFR2 for capture, and overall the VEGF dual dAb and aflibercept had a similar potency in both formats. Data are shown in Table 4 as the mean values from a set of 2–12 repeat experiments and in Fig. 2 for representation of data from one comparative experiment of the VEGF dual dAb with aflibercept.

**TABLE 4:** **Inhibition of VEGF-A_165_ binding to VEGFR1 and VEGFR2 by VEGF dual dAb and aflibercept** Data are the mean from *n* = 3 experiments, except bevacizumab (R1, *n* = 3; R2, *n* = 11) and ranibizumab (R2, *n* = 2) determined by MSD (numbers in parentheses represent S.D.).

Molecule	VEGFR1 IC_50_	VEGFR2 IC_50_
	*pm*	*pm*
Aflibercept	75 (10.2)	32 (3.5)
VEGF dual dAb	59 (11.8)	22 (2.7)
Bevacizumab	2204 (82.0)	2661 (1279)
Ranibizumab	ND*^a^*	1166 (124.9)

*^a^* ND, not determined.

##### Binding of VEGF Dual dAb Fc Region to FcRn, C1q, and Fcγ Receptors

The Fc region of VEGF dual dAb was tested for binding to FcRn, C1q, and Fcγ receptors in two formats: VEGF dual dAb alone and VEGF dual dAb in complex with human VEGF. VEGF dual dAb shows similar binding in the presence or absence of VEGF-165 to these effector proteins (Table 2). *K_D_* values could be derived for VEGF dual dAb (alone or in complex with human VEGF) in association with C1q and FcRn receptor (at pH 6, no binding occurred at pH 7.4), but for the FcγR receptors values could only be derived accurately for VEGF dual dAb alone, as binding in complex with VEGF was present but biphasic (data not shown).

##### Inhibition of VEGF-dependent Tubule Formation

**FIGURE 3.**
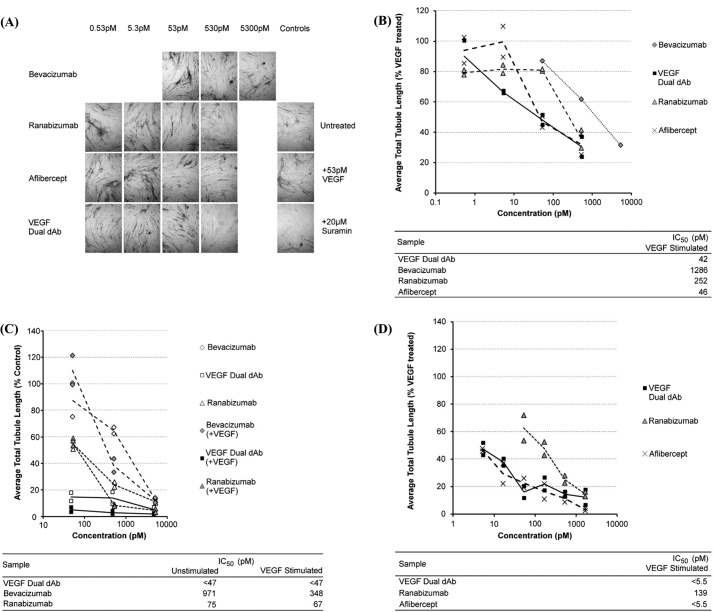
**Inhibition of VEGF-driven tubule-formation *in vitro* by VEGF dual dAb, aflibercept, ranibizumab, and bevacizumab.** *A*, representative tubule images from control and anti-VEGF-treated wells. Data were from one of three experiments; a single representative image of the three captured from each culture well using a 4× objective lens is shown. *B*, impact of anti-VEGF treatment on the total tubule length. Data were normalized to the 53 pm VEGF-treated positive control wells, and individual data points are plotted along with a line through the average value for each condition. Approximate IC_50_ values were determined from the inhibition curves. *C*, comparison of inhibitory activity of VEGF dual dAb, bevacizumab, and ranibizumab in untreated or VEGF-165 stimulated cultures, bevacizumab (♢) + VEGF (♦), VEGF dual dAb (□) + VEGF (■), and ranibizumab (▵) + VEGF (▴). *D*, comparison of inhibitory activity of VEGF dual dAb, aflibercept, and ranibizumab in VEGF-165-stimulated cultures, VEGF dual dAb + VEGF (■), ranibizumab + VEGF (▴), and aflibercept + VEGF (*X*). All conditions were tested in duplicate sample wells. Individual data points are plotted along with a line through the average value for each condition. Approximate IC_50_ values were determined from the inhibition curves.

*In vitro* tubule formation assays were used to investigate the potency of anti-VEGF molecules by preventing CD31-positive tubule formation as a model for angiogenesis. The addition of 2 ng/ml VEGF (53 pm) increased tubule formation 2–3-fold. Conversely, 20 μm suramin reduced tubule formation 5–10-fold. Using total tubule length as a measure of tubulogenesis, the IC_50_ of VEGF dual dAb was in the range 10–50 pm, but inter-experimental comparisons were not trivial due to differences in staining intensities. ELISA analysis data were comparable to image analysis, but variability due to background staining was greater (data not shown). Three independent experiments were performed in which VEGF dual dAb was compared with bevacizumab and ranibizumab, and two were performed in which VEGF dual dAb was compared with aflibercept with the relative activities of compounds being consistent in all. A representative experiment, in which all test compounds were compared, is shown in Fig. 3, *A* and *B*. The data displayed shows the effect of anti-VEGF treatments on tubule formation after 14 days of incubation of V2a cells treated with 53 pm exogenous VEGF assayed by image analysis of anti-CD31-stained cultures to determine total tubule length per image field (Fig. 3, *A* and *B*). In this assay VEGF dual dAb showed similar efficacy to aflibercept, ∼5-fold greater than ranibizumab and 25-fold greater than bevacizumab (Fig. 3*B*). Two further experiments confirming the ranking of molecules are shown in Fig. 3, *C* and *D*.

##### Stoichiometry of VEGF Dual dAb Binding to VEGF Determined by SEC-MALLS

**FIGURE 4.**
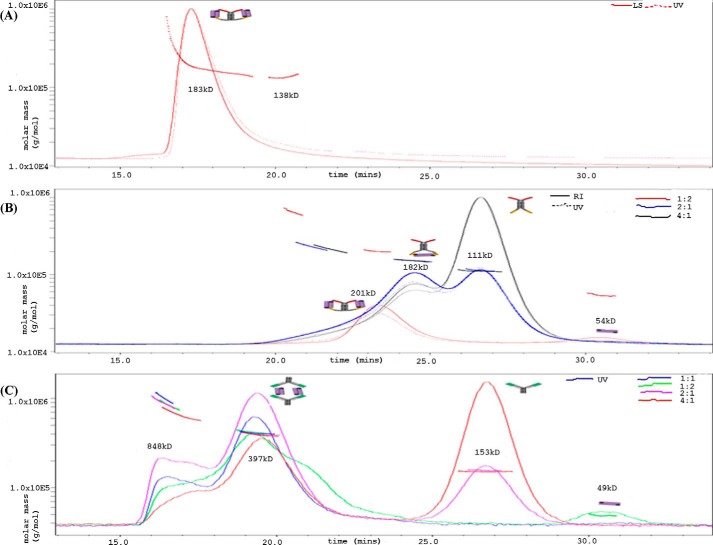
**SEC-MALLS analysis of anti-VEGF molecules with VEGF.** Retention time (min) is shown on the *x* axis, and molecular mass (g/mol) is shown on the *y* axis. The line across the peak is the molecular weight distribution assigned by MALLS across the peak resolved by SEC. The *solid*, *dash*, and *dotted lines* are signals from UV, refractive index, and light scattering, (molar mass calculated from concentration and light scattering), respectively. *A*, dual dAb in a 1:1 molar ratio complex with human VEGF_1–165_. Analysis is on the TSK3000. *B*, molar ratios of VEGF dual dAb-VEGF complex at 1:2, 2:1, and 4:1 were analyzed on the Superdex TM200 SWXL. *C*, shows molar ratios of bevacizumab-VEGF complex at 1:2, 1:1, 2:1, and 4:1 which were also analyzed on the TM200 SWXL and the SEC-MALLS profile compared with that of the VEGF dual dAb. Schematic models of the molecular interactions assigned to the major peaks are also shown based on Figs. 1*A*, 5*C*, and Fig. 6.

SEC-MALLS analysis of VEGF dual dAb in complex with VEGF_1–165_, mixed at a 1:1 molar ratio, is shown in Fig. 4*A*. The line across the peak is the molecular weight distribution assigned by MALLS across the peak resolved by SEC. These measurements are based on the Debye-Zimm equation, which determines the relationship between solute concentration, refractive index increment, Rayleigh excess ratio, incident wavelength, and molecular mass. The *solid*, *dashed*, and *dotted lines* are signals from ultraviolet (UV), refractive index, and light scattering used to determine molecular mass (calculated from concentration and light scattering), respectively. The signal from the three detectors (UV, refractive index, and light scattering) overlay well (a slight synchronization delay between UV and refractive index curves occurred; Fig. 4*A*), suggesting good quality homogeneous distribution of the complex. The arithmetic addition of the molecular masses (dual dAb: ∼109 kDa; VEGF dimer: ∼48 kDa, within the region of ±10–15% accuracy) suggests the predominant presence of a 1:2 binding stoichiometry with a single VEGF dual dAb molecule bound to two VEGF dimers. Slight trailing of the peak suggests nonspecific interaction of the complex with the stationary phase or a small proportion of weakly formed 1:1 complex. Sub- and super-stoichiometric complexes of the VEGF dual dAb to VEGF dimer at ratios of 1:2, 2:1, and 4:1 are shown in Fig. 4*B*. The data confirm the findings shown in Fig. 4*A*, with only complexes of the VEGF dual dAb and VEGF at 1:1 or 1:2 ratios of dual dAb-VEGF dimer being observed. This can be contrasted with data obtained concurrently with bevacizumab (148 kDa) and VEGF dimer at molar ratios of 1:1, 1:2, 2:1, and 4:1, where peaks corresponding to complexes of molar ratios of 2:2 (bevacizumab-VEGF dimer) or even larger aggregates predominate at all input ratios; Fig. 4*C*. This confirms previously published data with bevacizumab (35).

##### Crystal Structure of V_κ_·dAb and V_H_·dAb in Complex with Human VEGF

**FIGURE 5.**
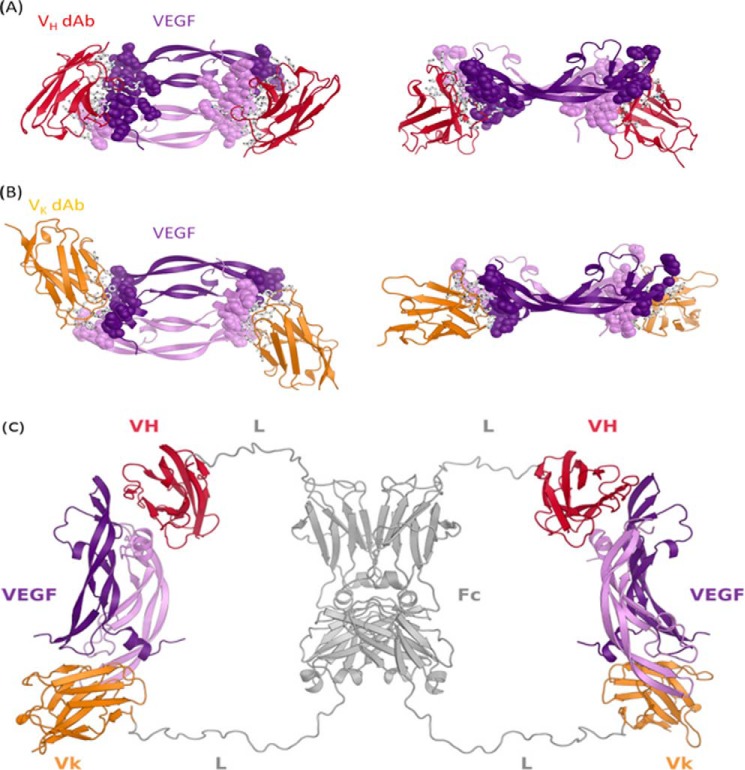
**X-ray crystal structure of the VEGF·dAb complexes and proposed structure of VEGF/VEGF dual dAb complex.** VEGF/V_κ_·dAb complex (*A*) and VEGF/V_H_·dAb complex (*B*) structures are depicted as secondary structure schematics with V_κ_·dAbs colored in *gold*, V_H_·dAbs colored in *red*, and the VEGF homodimer colored in *purple*. In *A* and *B*, side chains of VEGF epitope and dAb paratope residues are depicted as *spheres* and *sticks*, respectively. *C*, *in silico* built model showing how a dual-dAb molecule could engage VEGF. Modeling was based on the crystal structures of the VEGF-dAb complexes. Fc homodimer and linker regions are colored *gray*. Fc-fused V_H_ and V_κ_·dAb regions are in *red* and *gold*, respectively (*VH* = V_H_·dAb; *Vk* = V_κ_·dAb; *Fc* = hIgG Fc domain; *L* = peptide linker).

Fig. 5*A* shows the crystal structure of VEGF-specific V_κ_·dAb in complex with human VEGF_1–107_, and Fig. 5*B* shows the crystal structure of VEGF-specific V_H_·dAb in complex with human VEGF_1–107_. In both cases the complex appears to be a symmetric dimer of VEGF with two molecules of V_κ_·dAb or V_H_·dAb bound. V_κ_·dAb binding overlaps spatially with that of V_H_·dAb although orientation of the dAbs are clearly different in the two complexes. A more detailed view of interface between dAbs and VEGF_1–107_ shows that V_κ_·dAb interactions with VEGF are concentrated on complementarity determining regions, whereas the framework of V_H_·dAb is also involved in the paratope.

##### Molecular Model of VEGF Dual dAb Engagement with Human VEGF

The C-terminal Cα atoms of the V_H_·dAbs (these present at the N-terminal junction of the dAb with the Fc region in the full-length VEGF dual dAb) in the co-crystal structure (see supplemental Fig. 1 and Fig. 5*B*) are ∼81 Å apart.

Orientating the C-terminal residues of the dAbs against the N-terminal linkers of VEGF dual dAb shows that the distance between the N-terminal residues of the linkers is too short to enable them to attach to the dAbs when bound to VEGF unless the linkers are in an extremely extended conformation, the likelihood of which is very low.

**FIGURE 6.**
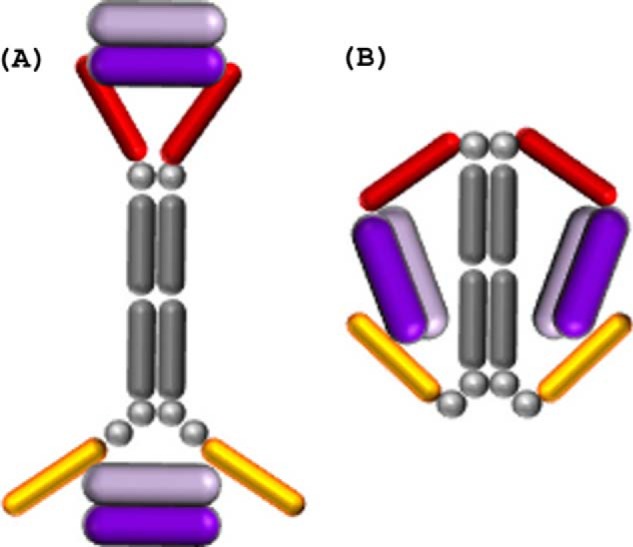
**Schematic of potential engagement mechanisms of the VEGF dual dAb molecule with two VEGF dimers.** *A*, end-on (where the two V_H_·dAbs fail to fully entrap the VEGF dimer). *B*, side-on; the VEGF dimer is displayed in *purple*, the V_H_·dAbs in *red*, the V_κ_·dAbs in *gold*, and the IgG Fc backbone and linkers in *gray*.

The N-terminal Cα atoms of the V_κ_·dAbs (these present at the C-terminal junction of the Fc region with the dAb in the full-length VEGF dual dAb) in the co-crystal structure (see supplemental Fig. 1 and Fig. 5*A*) are ∼70 Å apart. Orientating the N-terminal residues of the dAbs against the C-terminal linkers of VEGF dual dAb shows that the distance between the C-terminal residues can easily span the distance to enable them to attach to the dAbs when bound to VEGF. Such binding could explain one of the peaks in the SEC-MALLS analysis at high ratios of dual dAb-VEGF (see Fig. 4*B*). The molecular modeling suggests that the binding stoichiometry observed in the SEC-MALLS analysis is unlikely to be explained by a full “end-on” mode of interaction (Fig. 6*A*). For the dual dAb to engage a VEGF dimer at the top of the molecule, it would only be structurally favorable by sharing that engagement with the top of another dual dAb molecule (data not shown); if this were to occur with a VEGF dimer binding to the bottom end of the dual dAb molecules, then a complex of (1:2)_2_ should be formed (of approximate molecule mass of 370 kDa), but such a complex is not seen on SEC-MALLS analysis (Fig. 4, *A* and *B*). In contrast, with SEC-MALLS analysis of bevacizumab when interacting with VEGF where (1:1)_2_ complexes predominate (397 kDa) with even higher molecular weight aggregates (897 kDa (Fig. 4*C*)).

The N-terminal Cα atom of the V_κ_·dAb in the co-crystal structure and the C-terminal Cα atom of the V_H_·dAb on the opposite VEGF epitope in the co-crystal structure, when the structures are superimposed based on the VEGF molecules, are ∼77 Å apart. Orientating these Cα atoms against the appropriate C- and N-terminal linkers of VEGF dual dAb, as depicted in Fig. 5*C*, shows that the distance between the linker residues can easily span the distance to enable them to attach to the dAbs when bound to VEGF, making the “side-on” interaction (Fig. 6*B*) the most likely mode of engagement of VEGF dual dAb with human VEGF. This could fully explain the SEC-MALLS data (see Fig. 4, *A* and *B*). Part of these considerations are based upon the dAb interactions with VEGF_1–107_ in crystal structures but would be expected to hold for the dual dAb interaction with all the VEGF isoforms including VEGF-A_165_.

## Discussion

This report describes an example of a dual dAb molecular architecture potentially useful in the development of novel biopharmaceuticals and provides a mechanistic interpretation of the enhanced potency and binding capacity of an anti-VEGF dual dAb molecule.

Surface plasmon resonance experiments show that VEGF dual dAb binds to porcine, mouse, and human VEGF with similar affinities (see Table 1) and is able to bind different isoforms of human VEGF-A (VEGF-A_121_ and VEGF-A_165_) and mouse VEGF-164 with a higher affinity than ranibizumab, bevacizumab, and aflibercept in MSD format assays (see Table 3). VEGF dual dAb did not bind to other members of the human VEGF family, VEGF-B, -C, and -D, in contrast to aflibercept, which also binds both VEGF-A and VEGF-B (Ref. 21 and data not shown). VEGF dual dAb potency in inhibition of VEGF receptor binding assays was similar to that of aflibercept (although VEGF dual dAb IC_50_ was slightly lower) when preventing human VEGF-A_165_ binding to both VEGFR1 and VEGFR2 but was more potent than ranibizumab and bevacizumab (see Table 4 and Fig. 2). The binding and neutralization of VEGF has recently been compared for the current anti-VEGF biologics in clinical use (21). Aflibercept (Eylea/VEGF Trap) is consistently able to outperform both ranibizumab (Lucentis) and bevacizumab (Avastin) in a range of *in vitro* assays including VEGF ligand binding (21). However, efforts to use SPR antigen binding values in 1:1 models as a way to rank binding capacity has been found to be problematic in an analysis of ranibizumab, bevacizumab, and aflibercept (36). If the molecule has more than one VEGF binding site, as is the case with the VEGF dual dAb, one has to be very careful in interpreting comparative *in vitro* data with other VEGF inhibitors with only a single VEGF binding site. Normalized VEGF molecule binding values may, as with SPR, take an average of the two VEGF dimer binding sites and do not score for improved molecule stoichiometry. Such comparisons are hard to make with respect to other published *in vitro* data as the type of assays vary in key subtle details across different publications (21).

Characterization of the Fc domain of VEGF dual dAb was also carried out with binding to C1q (subcomponent of the complement C1 complex), neonatal Fc receptor FcRn, and a panel of human Fcγ receptors. This was analyzed for VEGF dual dAb alone or complexed with human VEGF. The *K_D_* values obtained from the experiments with C1q shows that VEGF dual dAb alone or in complex with human VEGF binds to C1q with similar affinity. A similar pattern was observed with binding to FcRn. As expected, no FcRn binding was obtained for any of the samples at pH 7.4; however, at pH 6 VEGF dual dAb both alone and in complex with VEGF bound to FcRn with similar affinities as shown by the *K_D_* values obtained from these experiments. Binding of VEGF dual dAb alone or in complex with human VEGF was observed with all human Fcγ receptors tested; however, binding of the VEGF dual dAb in complex with VEGF generated biphasic curves, suggesting a possible oligomerization of the VEGF dual dAb when in complex with the VEGF under certain conditions, especially when accessing these large Fcγ receptors.

Protein engineering studies of mAbs suggest that the human IgG1 interaction site for C1q/C1 is localized to the hinge proximal region of the CH2 domain (17). This site is preserved in the VEGF dual dAb molecule and is likely accessible to C1q binding in the presence of bound VEGF (Table 2). The interaction site on the IgG1-Fc of mAbs with the human FcRn is at the CH2/CH3 interface (17, 19), and this region is also present in the VEGF dual dAb molecule with FcRn binding clearly maintained in the presence of bound VEGF, as binding of VEGF dual dAb to FcRn is similar in the presence and absence of bound VEGF.

SEC-MALLS is a powerful tool to measure molecular weight distribution of molecules in solution. In this study SEC was used to separate complexes of VEGF dual dAb and VEGF based on their hydrodynamic volume. Light scattering analysis was then performed using MALLS on the separated fractions to help determine molecular mass and deduce the most likely stoichiometry of VEGF dual dAb binding to antigen in the presence of comparable or excess molar amounts of VEGF. The single ∼183–201 kDa complex (see Fig. 4, *A* and *B*) is close (within ±10–15% error) to the combined molecular mass of the VEGF dual dAb (∼109 kDa) and two VEGF_1–165_ dimers (2 x ∼48.0 kDa). The data suggest that each VEGF dual dAb molecule engages two VEGF dimers and that this is the major species formed when the ratios of molecule to VEGF are varied in favor of VEGF (see Fig. 4, *A* and *B*). A previous study has compared the stoichiometry of interaction of both aflibercept (Eylea/VEGF Trap) and bevacizumab with VEGF using SEC-MALLS (35). Aflibercept was shown to form a 1:1 complex with a single VEGF dimer, but in contrast bevacizumab was found to form both a 2:2 complex and a heterogeneous mix of very high molecular mass complexes or aggregates that were suggested to be the multimeric immune complexes seen when bevacizumab is dosed systemically *in vivo* (35). In this study we were able to confirm that bevacizumab and VEGF form high molecular mass complexes (see Fig. 4*C*). The SEC-MALLS analysis of VEGF dual dAb in complex with VEGF does not show high molecular mass aggregates (Fig. 4, *A* and *B*) but at elevated VEGF levels generates a single complex similar to aflibercept, with VEGF dual dAb “trapping” two VEGF dimers instead of one as in the case of aflibercept.

Fig. 1 illustrates that although most published anti-VEGF antibodies or antibody fragments all bind at a ratio of 2 molecules per dimer of VEGF (2:2), the binding epitope and paratopes vary considerably among the complexes. Interestingly bevacizumab and ranibizumab, which are both human-specific, share a similar epitope when compared with G6, which was found by phage display, and shows murine and human cross-species activity. The figure represents previously published data (34, 37, 38).

Complexes of VEGF_1–107_ bound to V_H_·dAb and V_κ_·dAbs were generated and successfully crystallized. After determination of the x-ray crystal structure of the two distinct complexes, we observed V_H_·dAb and V_κ_·dAb binding to similar epitopes on VEGF when comparing the two structures. However, this is not surprising as both dAbs were independently capable of inhibiting VEGF binding to both VEGFR1 and -R2, the activity for which lies within a short linear amino acid stretch of VEGF (see Fig. 1 and data not shown, Ref. 39).

When considering the end-on mode of engagement (Fig. 6*A*), *in silico* modeling studies demonstrate that it is highly unlikely that the two V_H_·dAbs at the N terminus of VEGF dual dAb could bind simultaneously to a single VEGF dimer, although it is possible for the V_κ_·dAbs at the C terminus to do so (see Fig. 4*B*, 2:1 and 4:1 input molar ratios of dual dAb-VEGF). The predictions are supported by SPR studies of the “top half” (V_H_·dAb fused with IgG1 Fc in the absence of C-terminal V_κ_·dAbs) and “bottom half” (IgG1 Fc alone fused to V_κ_·dAbs) of the VEGF dual dAb molecule binding to VEGF. The top half showed reduced affinity, largely due to a more rapid dissociation rate, whereas the bottom half showed very similar VEGF affinity to the full VEGF dual dAb (data not shown).^5^ When considering the side-on (Fig. 6*B*) mode of engagement, *in silico* modeling studies demonstrate that it is possible for a single V_H_·dAb at the N terminus and a single V_κ_·dAb at the C terminus of VEGF dual dAb to bind to one VEGF dimer simultaneously. It can reasonably be assumed that this would be possible on both halves of an intact molecule of VEGF dual dAb; therefore, we predict that a single molecule of VEGF dual dAb is most likely to bind to two VEGF dimers via the side on mode of engagement, and this is consistent with the observed SEC-MALLS data. It is also consistent with the single high affinity SPR binding event of VEGF for the VEGF dual dAb as a mean of two “identical” VEGF binding sites (Table 1). The definition of the dAb binding sites on VEGF helped to predict the side on engagement model as a likely novel mechanism of VEGF binding for the dual dAb. The generation of a molecule with the capacity to “trap” two VEGF dimers offers a potential potency advantage at low VEGF dual dAb concentrations in relation to VEGF levels.

Parallel work aims to develop the VEGF dual dAb in a compatible slow-release delivery system and to evaluate its efficacy *in vivo*,^3^ the ultimate aim being to extend the treatment interval of VEGF driven ocular angiogenic disorders that show vascular leak such as wet age-related macular degeneration, diabetic macular edema, retinal vein occlusion and potentially cancers such as glioblastoma with a high vascular dependence (2, 4).

## Author Contributions

A. W., M. S., and I. C. conceived and coordinated the study and wrote the paper. M. M., V. C., and J. K. selected, constructed, and prepared the VEGF dual dAb molecules. N. T. and C.-M. T. designed, performed, and analyzed the experiments shown in Tables 1–4 and Fig. 2. C.-W. C. designed Fig. 1. C.-W. C., M. N., and T. B. designed, performed, and analyzed the experiments shown in supplemental Table 1 and Fig. 1. G. J. designed, performed, and analyzed the experiments shown in Fig. 3. M. B., J. K., and A. W. designed, performed, and analyzed the experiments shown in Fig. 4. A. L., C.-W. C, T. B, A. W., and I. C. provided input to the generation and preparation of Figs. 5 and 6. All authors reviewed the results and approved the final version of the manuscript.

## Supplementary Material

Supplemental Data

## Footnotes

3 P. Adamson, T. Wilde, E. Dobrzynski, C. Sychterz, R. Polsky, E. Kurali, R. Haworth, C. M. Tang, J. Korczynska, F. Cook, I. Papanicolaou, L. Tsikna, C. Roberts, Z., Hughes-Thomas, J. Walford, D. Gibson, J. Warrack, J. Smal, J. Van der Zwann, R. Verrijk, P. E. Miller, T. M. Nork, J. Prusakiewicz, T. Streit, S. Sorden, C. Struble, B. Christian, and I. Catchpole, manuscript submitted for publication.

5 A. Walker, C.-W. Chung, M. Neu, M. Burman, T. Batuwangala, G. Jones, C.-M. Tang, M. Steward, M. Mullin, N. Tournier, A. Lewis, J. Korczynska, V. Chung, and I. Catchpole, unpublished data.

## Footnotes

4 The abbreviations used are:

VEGFR: VEGF receptor
dAb: domain antibody
MSD: meso-scale discovery
SPR: surface plasmon resonance
SEC-MALLS: size exclusion chromatography multi-angle laser light scattering
Bis-Tris: 2-[bis(2-hydroxyethyl)amino]-2-(hydroxymethyl)propane-1,3-diol
Bistris propane: 1,3-bis[tris(hydroxymethyl)methylamino]propane.
