# Supplementary material for: Novel Interaction Mechanism of a Domain Antibody-based Inhibitor of Human Vascular Endothelial Growth Factor with Greater Potency than Ranibizumab and Bevacizumab and Improved Capacity over Aflibercept
Source: J Biol Chem. 2016 Jan 4;291(11):5500–11. doi: 10.1074/jbc.M115.691162 (PMC4786692; doi:10.1074/jbc.M115.691162)
Supplement: Supplemental Data [file supp_291_11_5500__index.html]

Novel interaction mechanism of a domain antibody based inhibitor of human vascular endothelial growth factor with greater potency than ranibizumab and bevacizumab and improved capacity over aflibercept. — Novel Interaction Mechanism of a Domain Antibody-based Inhibitor of Human Vascular Endothelial Growth Factor with Greater Potency than Ranibizumab and Bevacizumab and Improved Capacity over Aflibercept — Novel Interaction Mechanism of a dAb-based Inhibitor — Supplemental Data 

# Novel Interaction Mechanism of a Domain Antibody-based Inhibitor of Human Vascular Endothelial Growth Factor with Greater Potency than Ranibizumab and Bevacizumab and Improved Capacity over Aflibercept

## Supplemental Data

- Revised supplemental data (.pdf, 868 KB) - Revised supplemental data
