# Supplementary material for: Novel Interaction Mechanism of a Domain Antibody-based Inhibitor of Human Vascular Endothelial Growth Factor with Greater Potency than Ranibizumab and Bevacizumab and Improved Capacity over Aflibercept
Source: J Biol Chem. 2016 Jan 4;291(11):5500–11. doi: 10.1074/jbc.M115.691162 (PMC4786692; doi:10.1074/jbc.M115.691162)
Supplement: Supplemental Data [file 10.1074_M115.691162_jbc.M115.691162-1.pdf]

Novel interaction mechanism of a domain antibody based inhibitor of human vascular endothelial growth factor with greater potency than ranibizumab and bevacizumab and improved capacity over aflibercept.

Adam Walker[1], Chun-wa Chung[2], Margaret Neu[2], Manish Burman[1], Thil Batuwangala[1], Gavin Jones[1], Chi-Man Tang[1*], Michael Steward[1], Michael Mullin[3], Nadia Tournier[3], Alan Lewis[3], Justyna Korczynska[1,3], Vicky Chung[4], Ian Catchpole[1#]

SUPPLEMENTAL DATA.

SI Figure 1: Sample electron density maps of the V_k dAb/VEGF-107 interaction, (**A**) V_k dAb L chain and (**B**) M chain and V_H dAb/VEGF-107 complexes, (**C**) V_H dAb L chain and (**D**) M chain.

(A)

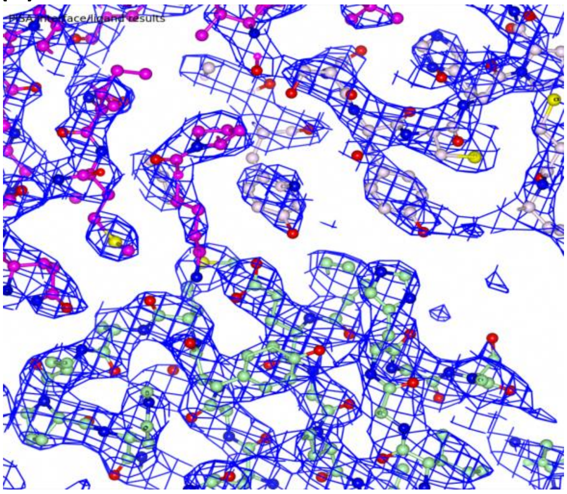

2fofc (dark blue contoured at 1.5sigma) electron density map of interface region of Vk/VEGF focused on L chain of Vk dAb

(V Chain of VEGF(pink); W chain of VEGF (magenta); L chain of Vk dAb (light green) ; M chain of Vk dAb (green)

(B)

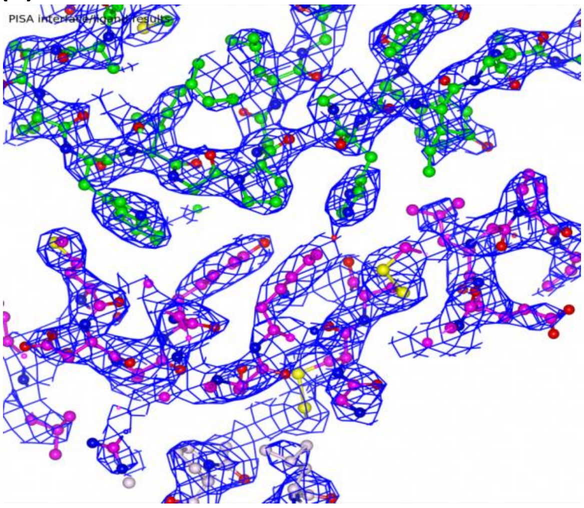

2fofc (dark blue contoured at 1.5sigma) electron density map of interface region of Vk/VEGF focused on M chain of Vk dAb

(V Chain of VEGF(pink); W chain of VEGF (magenta); L chain of Vk dAb (light green) ; M chain of Vk dAb (green)

(C)

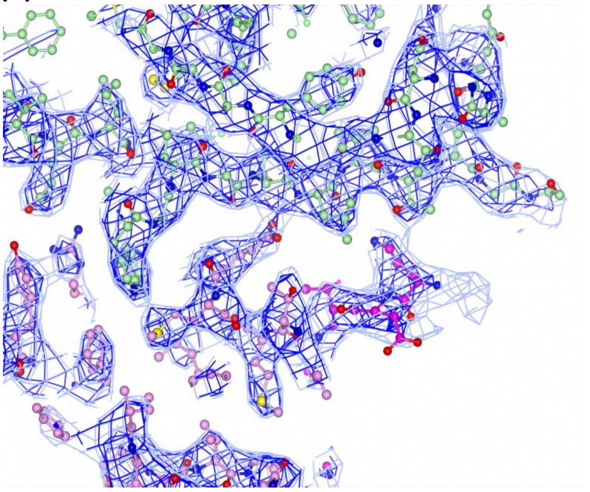

2fofc electron density map of interface region of V_H/VEGF focused on M chain of V_H dAb

(2fofc map dark blue contoured at 1.5sigma, light blue at 1sigma)

(V Chain of VEGF(pink); W chain of VEGF (magenta); A chain of V_H dAb (light green) ; B chain of V_H dAb (green)

(D)

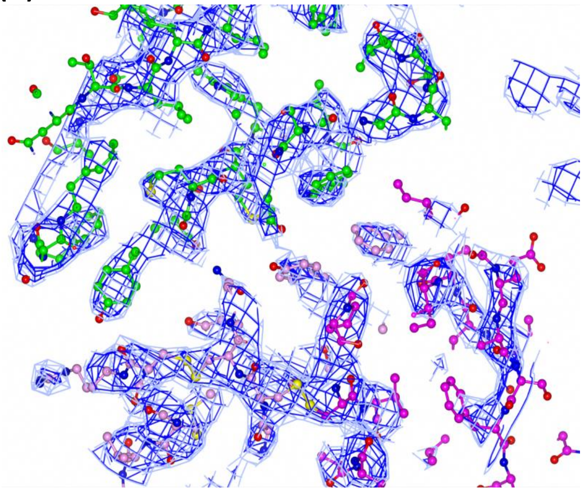

2fofc electron density map of interface region of V_H/VEGF focused on M chain of V_H dAb

(2fofc map dark blue contoured at 1.5sigma, light blue at 1sigma)

V Chain of VEGF(pink); W chain of VEGF (magenta); A chain of V_H dAb (light green) ; B chain of V_H dAb (green

SI Table 1: Statistics for Xray data collection and refinement

(collection on a single crystal)	**V_K dAb: VEGF**	**V_H dAb: VEGF**
**Data collection ***		
Space group	I222	C2
Cell dimensions		
a, *b*, *c* (Å)	61.22, 147.13, 175.17	107.11, 130.40, 81.18
α, β, γ (°)	90.000, 90.000, 90.000	90.000, 106.53, 90.000
Resolution (Å)	73.56-2.70 (2.85-2.70)	51.34-3.45 (3.64-3.45)
R_{merge}	0.088 (0.482)	0.0112 (0.439)
$I/\sigma I$	18.5 (3.8)	7.8 (2.7)
Completeness (%)	99.5 (100.0)	99.4 (99.9)
Redundancy	6.6 (6.8)	3.8 (3.7)
**Refinement**		
Resolution (Å)	73.56 -2.70	51.34 -3.45
No. reflections	145182 (21768)	52467 (7842)
No. uniq reflections	22056 (3181)	14048 (2066)
$R_{work/}$ R_{free}	0.205/0.261	0.214/0.262
No. atoms	3329	5024
Protein	3256	5024
Ligand/ion	0/0	0/0
Water	73	0
B-factors		
Protein	74.625	110.313
Ligand/ion	0/0	0/0
Water	63.849	/
R.m.s deviations		
Bond lengths (Å)	0.007	0.004
Bond angles (º)	1.202	0.967
**Ramachandran plot #**		
Most favoured regions [%]	91	88.3
Additional allowed regions	9	11.7
Generously allowed regions	0	0
Disallowed regions [%]	0	0

- Highest resolution shell is shown in parenthesis

Calculated by Procheck
